# Inhaled milrinone in cardiac surgical patients: pharmacokinetic and pharmacodynamic exploration

**DOI:** 10.1038/s41598-023-29945-7

**Published:** 2023-03-02

**Authors:** Anne Quynh-Nhu Nguyen, André Y. Denault, Yves Théoret, France Varin

**Affiliations:** 1 Faculty of Pharmacy, Université de Montréal, 2940 Chemin de la Polytechnique, Montreal, QC H3T 1J4 Canada; 2 Department of Anesthesiology and Critical Care Division, Montreal Heart Institute, Université de Montréal, 5000 Belanger Street, Montreal, QC H1T 1C8 Canada; 3 Clinical Pharmacology Unit, CHU Sainte-Justine, Montreal, Canada

**Keywords:** Drug delivery, Cardiovascular biology, Drug discovery, Physiology, Cardiology

## Abstract

Mean arterial pressure to mean pulmonary arterial pressure ratio (mAP/mPAP) has been identified as a strong predictor of perioperative complications in cardiac surgery. We therefore investigated the pharmacokinetic/pharmacodynamic (PK/PD) relationship of inhaled milrinone in these patients using this ratio (R) as a PD marker. Following approval by the ethics and research committee and informed consent, we performed the following experiment. Before initiation of cardiopulmonary bypass in 28 pulmonary hypertensive patients scheduled for cardiac surgery, milrinone (5 mg) was nebulized, plasma concentrations measured (up to 10 h) and compartmental PK analysis carried out. Baseline (R_0_) and peak (R_max_) ratios as well as magnitude of peak response (∆_Rmax-R0_) were measured. During inhalation, individual area under effect-time (AUEC) and plasma concentration–time (AUC) curves were correlated. Potential relationships between PD markers and difficult separation from bypass (DSB) were explored. In this study, we observed that milrinone peak concentrations (41–189 ng ml^−1^) and Δ_Rmax-R0_ (− 0.12–1.5) were obtained at the end of inhalation (10–30 min). Mean PK parameters agreed with intravenous milrinone published data after correction for the estimated inhaled dose. Paired comparisons yielded a statistically significant increase between R_0_ and R_max_ (mean difference, 0.58: 95% CI 0.43–0.73; *P* < 0.001). Individual AUEC correlated with AUC (r = 0.3890, r^2^ = 0.1513; *P* = 0.045); significance increased after exclusion of non-responders (r = 4787, r^2^ = 0.2292; *P* = 0.024). Individual AUEC correlated with ∆R_max-R0_ (r = 5973, r^2^ = 0.3568; *P* = 0.001). Both ∆R_max-R0_ (*P* = 0.009) and CPB duration (*P* < 0.001) were identified as predictors of DSB. In conclusion, both magnitude of peak response of the mAP/mPAP ratio and CPB duration were associated with DSB.

## Introduction

Cardiopulmonary bypass (CPB) is performed during cardiac surgery in order to maintain perfusion and oxygenation to all organs, besides the heart and lungs. Hemodynamic complications associated with difficult separation from bypass (DSB)^1^ represent a leading cause of mortality in cardiac surgery^2^. Pulmonary hypertension (PH) that can lead to right ventricular dysfunction was identified as one of the most important hemodynamic predictor and risk factor for DSB^3,4^. Amongst other hemodynamic parameters used in cardiac surgery, the mean artery pressure (mAP) to mean pulmonary artery pressure (mPAP) ratio has proved to be a predictor of perioperative complications^5–10^. In addition, the successful effect of inhaled therapy is expected to be associated with an increase in mAP/mPAP ratio and normalization of right ventricular function^11–13^. The mAP/mPAP ratio (R) remains unchanged following induction of general anesthesia^5^ and correlates with the eccentricity index which reflects the interventricular septal deformation in response to PH^14^.

Intravenous milrinone is commonly used for the treatment of PH when DSB occurs at the end of cardiac surgery^15–18^. An important drawback of intravenous milrinone is its association with systemic hypotension^19–21^. Therefore, inhalation has been proposed as an alternative route of administration for milrinone^22–24^. Inhaled milrinone administered before CPB has also been proposed as having a protective effect during cardiac surgery^11,25–27^ and a potential to facilitate separation from CPB in patients with PH^28^. In a clinical trial report, mAP/mPAP ratio was used as a pharmacodynamic (PD) marker to explore the relationship between milrinone concentration and effect exposures during inhalation period^29^. A relationship was found, but inhaled milrinone did not prove to facilitate separation from CPB. In that study, limited blood sampling did not allow full characterization of a pharmacokinetic (PK) and PD profile and, most importantly, a good estimation of the time point corresponding to peak concentration.

This report on inhaled milrinone will present results obtained from a full-scale PK/PD study in cardiac patients undergoing CPB having two major objectives: characterization of inhaled milrinone PKs and exploration of the concentration-effect relationship. As exploratory objectives, we wanted to identify potential predictors of DSB.

## Results

**Figure 1:**
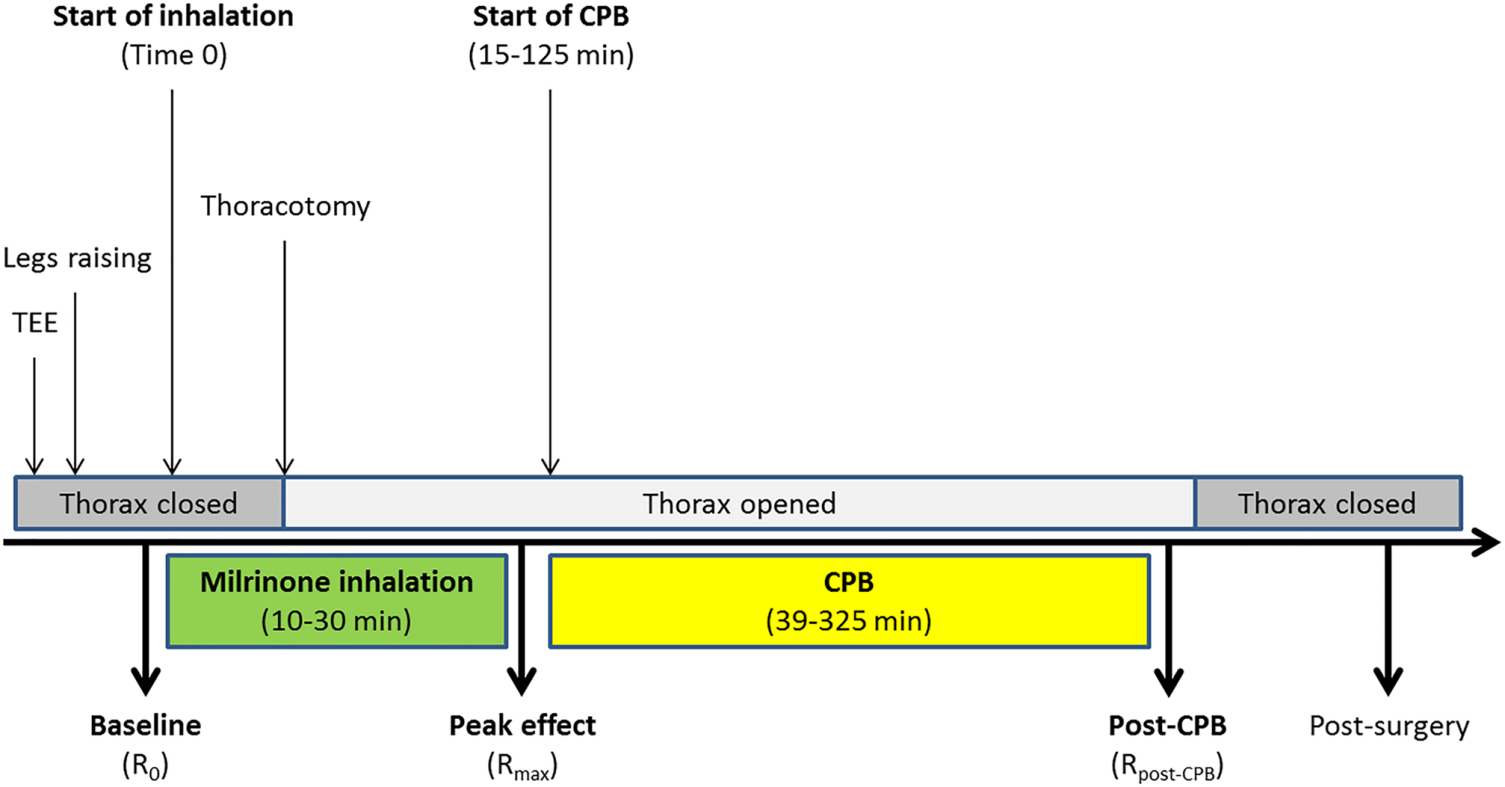
Important events and cut-off times used for data analysis on a typical cardiac surgical procedure time flow chart. *CPB* cardiopulmonary bypass, *R*_*0*_ baseline mAP/mPAP ratio, *R*_*max*_ peak mAP/mPAP ratio, *R*_*post-CPB*_ post-CPB mAP/mPAP ratio, *TEE* transesophageal echocardiographic exam.

**Figure 2:**
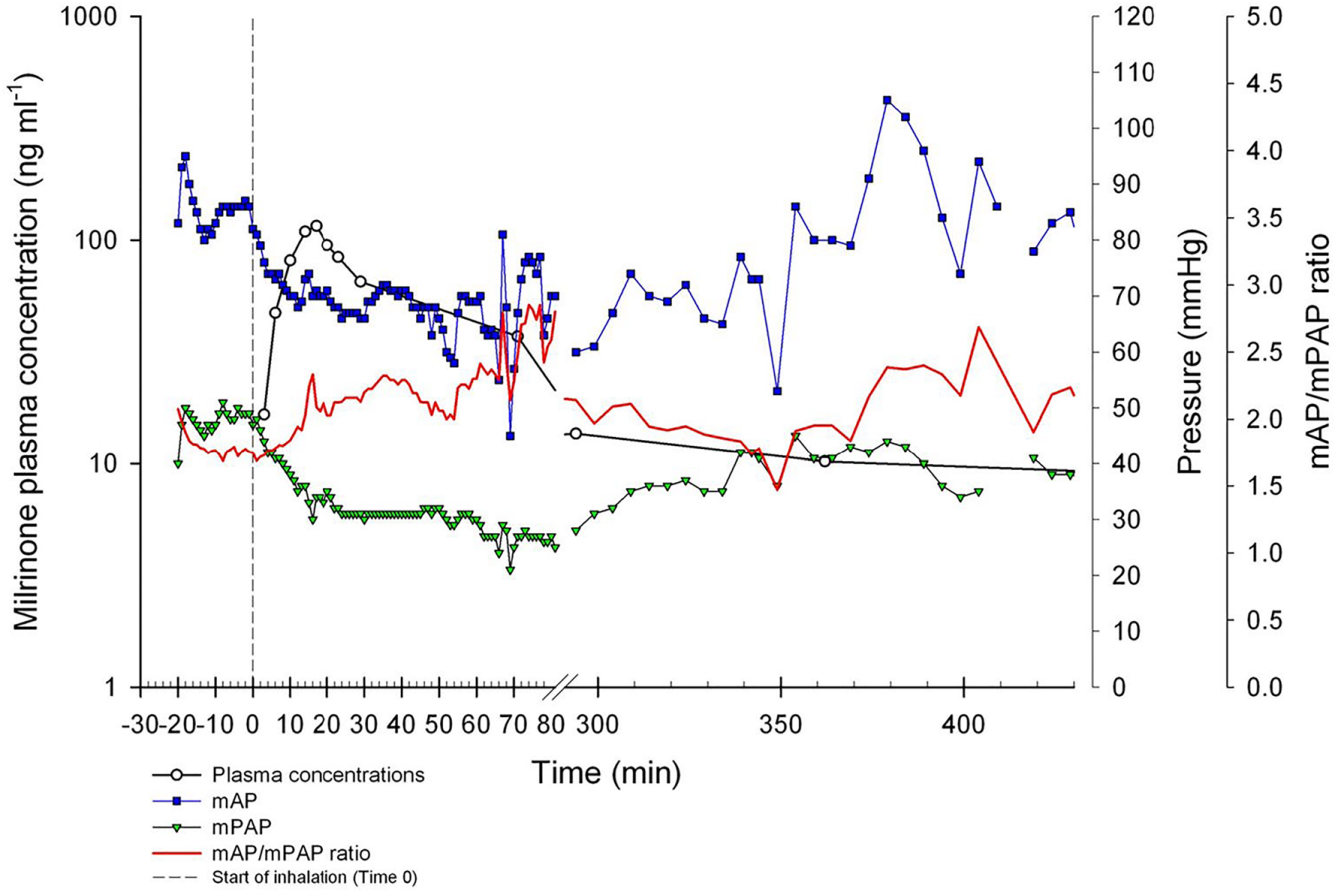
Typical plasma concentration–time profile and effect-time profile for one patient. *mAP* mean arterial pressure, *mPAP* mean pulmonary arterial pressure.

A total of 28 patients were recruited. Demographic and perioperative data are shown in Table 1. Important events and cutoff times used during data analysis are presented on a typical cardiac surgical procedure flowchart (Fig. 1). An example of PK and PD profiles obtained in a responder is shown in Fig. 2.

**Table 1:** Patient demographic and perioperative data.

	n ± sd
Gender	
Female	12
Male	16
Age (year)	67 ± 10
Weight (kg)	72 ± 10
Height (cm)	165 ± 7
Preoperative EuroSCORE II	8.0 ± 8.7
Parsonnet score	36 ± 10
SPAP at recruitment (mmHg)	67 ± 17
Creatinine clearance	61 ± 26
Renal impairment	
Normal	4
Moderate	12
Severe	12
Type of surgical procedure	
CABG	1
Single valve	12
Complex	13
Other (ASD, maze)	2
Milrinone nebulization time (min)	17 ± 6
CPB duration (min)	116 ± 72
DSB	
Yes	10
No	17

*ASD* atrial septal defect, *CABG* coronary artery bypass graft, *CPB* cardiopulmonary bypass, *DSB* difficult separation from CPB, *SPAP* systolic pulmonary artery pressure, *sd* standard deviation.

### Pharmacokinetic study

#### PK sampling

**Figure 3:**
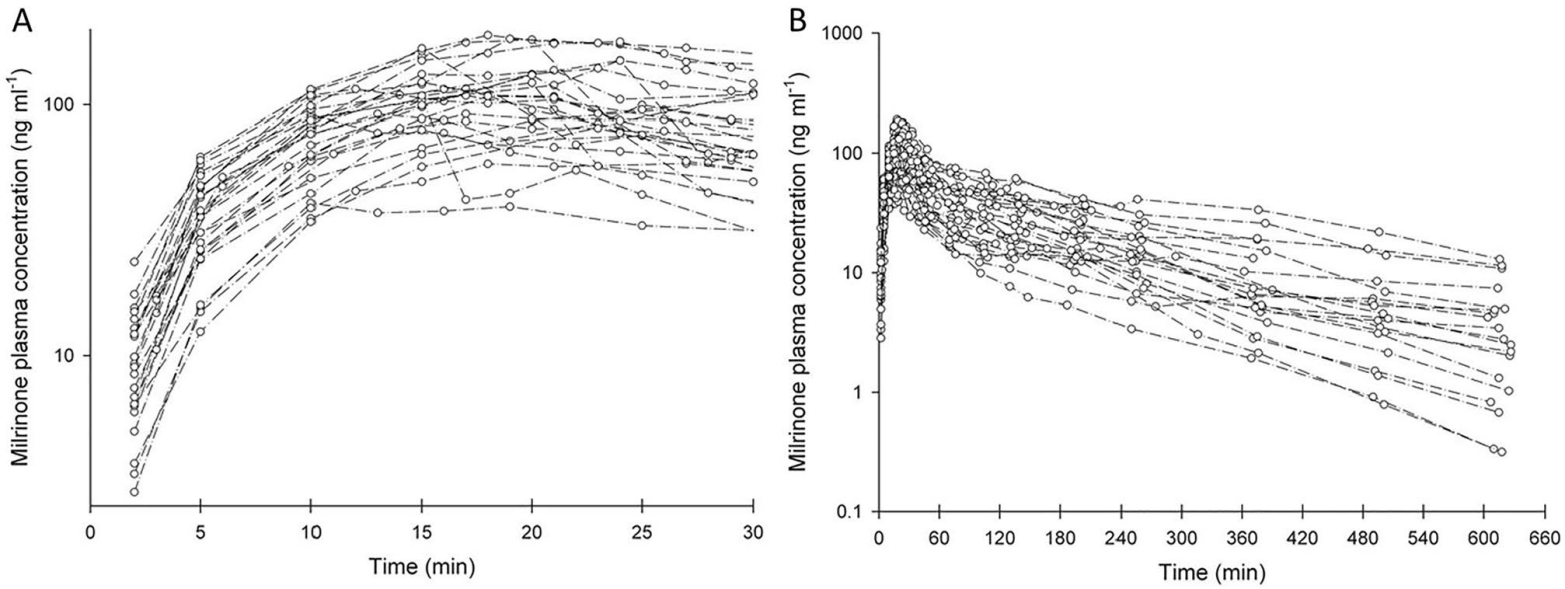
Individual milrinone plasma concentration–time profiles during inhalation (10–30 min) (**A**) and overall until 600 min after termination of inhalation (**B**) (n = 28).

Individual milrinone plasma concentration–time profiles during inhalation (A) (10–30 min) and overall from 0 to 10 h after inhalation (B) are presented in Fig. 3. One patient was scheduled to receive elective cardiac surgery but did not undergo CPB (intraoperative decision) and was only considered for PK analysis during the inhalation period. Milrinone average treatment time was 17 ± 6 min, ranging from 10 to 30 min. Mean nebulization rate was 0.086 ± 0.044 mg min^−1^ (0.021–0.237 mg min^−1^). Overall, C_max_ values ranged between 41 and 189 ng ml^−1^ and were observed at the end of inhalation. In all 28 patients’ plasma concentrations were quantifiable up to 10 h after termination of inhalation.

#### Inhaled dose

Mean percentages of dose recovered from individual in vivo results combined with previously determined mean in vitro results indicated almost complete recovery (95.3% ± 10.7%) of milrinone nominal dose (5 mg) (Supplementary Table S1). In patients (n = 15), mean cumulative amount of milrinone excreted in urine over a 24-h period was 1.29 ± 0.41 mg (25.8% of the 5 mg nominal dose) while the mean estimated inhaled dose using the back-calculated approach was 1.52 ± 0.32 mg (30.5%). As corresponding values did not differ (mean difference, 0.23 mg: 95% CI − 0.06 to 0.53; *P* = 0.112), this back-calculated approach for the estimation of the inhaled dose was considered acceptable for milrinone and used for PK analysis in all patients (n = 28).

#### PK analysis

Mean PK parameter estimates obtained after fitting to data a two-compartment model (1/ŷ) with a zero-order input rate during the nebulization period are presented in Table 2. Mean terminal elimination half-life was 154 ± 17 min. The milrinone systemic exposure or AUC was found to be inversely proportional to the nebulization rate (r = 0.4728, r^2^ = 0.2235; *P* = 0.011). The non-compartmental analysis is summarized in Supplementary Table S2.

**Table 2:** Milrinone PK parameters after fitting a two-compartment model with zero-order input to individual data.

V_c_/F (L kg^−1^)	V_ss_/F (L kg^−1^)	Cl/F (L h^−1^ kg^−1^)	A	B	α (min^−1^)	β (min^−1^)
0.12 ± 0.06	0.39 ± 0.25	0.11 ± 0.05	162 ± 89	42 ± 20	0.0944 ± 0.0984	0.0042 ± 0.0015

All values are mean (standard deviation). *A* coefficient of biexponential equation describing distribution curve, *B* coefficient of biexponential equation describing elimination curve, *Cl* total body clearance, *F* bioavailability, *PK* pharmacokinetic, *V*_*c*_ apparent volume of distribution of central compartment, *V*_*ss*_ apparent volume of distribution at steady-state, *α* distribution rate constant, *β* elimination rate constant.

### Pharmacodynamic study

#### PD markers

**Figure 4:**
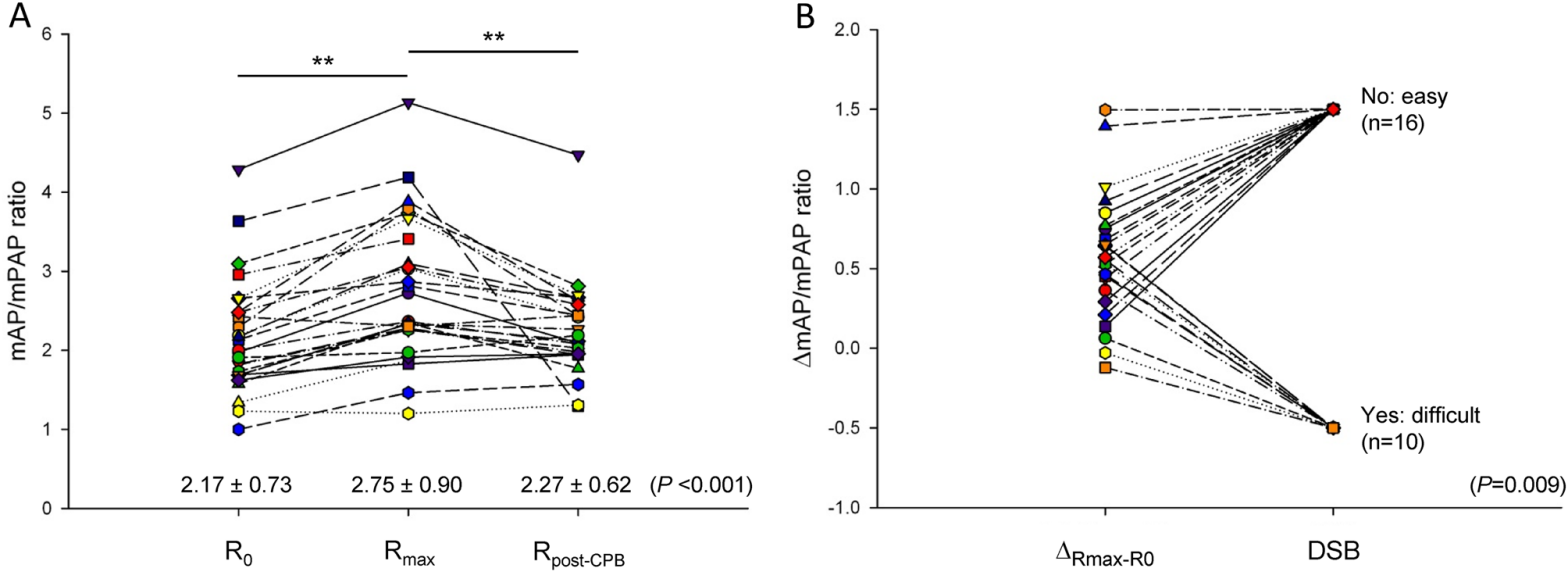
Association between R_0_ (n = 27), R_max_ (n = 27) and R_post-CPB_ (n = 25) using one-way repeated measures analysis of variance (ANOVA) (**A**) and association between ∆_Rmax-R0_ and DSB (clinical endpoint) using simple logistic regression (**B**). (Pulmonary artery catheter unavailable in one patient). Mean ± SD **P < 0.001. *DSB* difficult separation from bypass, *mAP* mean arterial pressure, *mPAP* mean pulmonary arterial pressure, *R*_*0*_ baseline mAP/mPAP ratio, *R*_*max*_ peak mAP/mPAP ratio, *R*_*post-CPB*_ post-CPB mAP/mPAP ratio, *∆*_*Rmax-R0*_ magnitude of peak response.

One patient was not considered for PD analysis after unsuccessful Swan-Ganz installation. For all patients, paired comparisons between R_0_, R_max_ and R_post-CPB_ yielded a statistically significant increase between R_0_ and R_max_ (mean difference, 0.58: 95% CI 0.43 to 0.73; *P* < 0.001) representing a mean increase from baseline of 26.6% but not between R_0_ and R_post-CPB_ (mean difference, 0.10: 95% CI − 0.12 to 0.33; *P* = 0.358) with a less substantial mean increase from baseline of 4.7% (Fig. 4A). Using a simple logistic regression, ∆_Rmax-R0_ was found to be directly related to the clinical endpoint DSB (*P* = 0.009) (Fig. 4B). When patients were categorized according to the occurrence of DSB, ∆_Rmax-R0_ was 0.37 (17.4%) in patients with DSB compared to 0.71 (31.3%) in patients without DSB (mean difference, 0.34: 95% CI 0.07 to 0.61; *P* = 0.015).

#### PK/PD analysis

**Figure 5:**
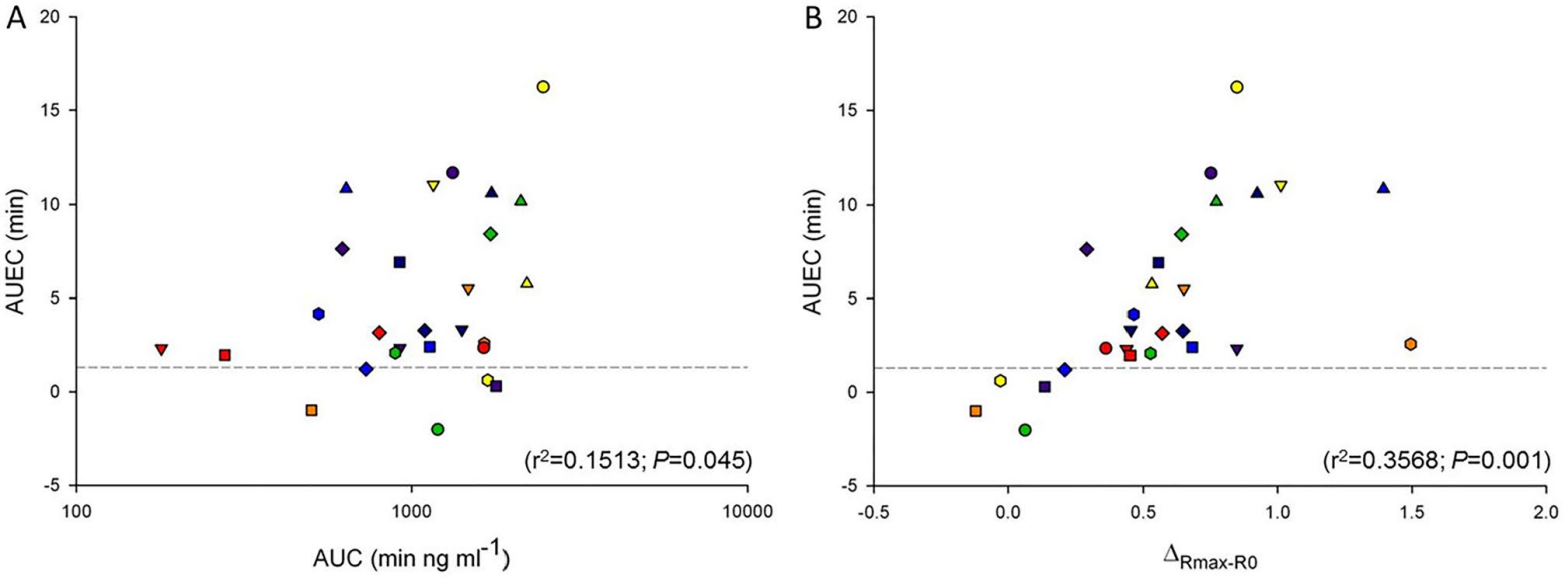
Relationship between AUEC and AUC (**A**) and relationship between AUEC and ∆_Rmax-R0_ (**B**) for the inhalation period (10–30 min) using linear regression models (n = 27). *AUC* area under the plasma concentration–time curve, *AUEC* area under the effect-time curve, *∆*_*Rmax-R0*_ magnitude of peak response.

During the inhalation period, the relationship between AUEC and AUC was best explained by a linear regression model (r = 0.3890, r^2^ = 0.1513; *P* = 0.045) (Fig. 5A). The minimum threshold for therapeutic response in patients, i.e. the AUEC-intercept, was estimated as 1.387. Accordingly, 22 patients out of 27 were considered as responders. The exclusion of non-responders resulted in an improvement of this correlation (r = 4787, r^2^ = 0.2292; *P* = 0.024). Finally, the overall exposure to pharmacological response, AUEC, was also correlated with ∆_Rmax-R0_ (r = 5973, r^2^ = 0.3568; *P* = 0.001) (Fig. 5B).

#### Clinical endpoint

The variables retained during forward analysis are presented in Table 3.

**Table 3:** Determination of explanatory variables in a logistic model for DSB.

Model variables	n	− 2LL	LRT *P*-value	∆ (− 2LL)
STEP 1				
DSB + effect of EuroSCORE II	27	31.223	0.037	
DSB + effect of R_0_	26	34.129	0.472	
DSB + effect of R_max_	26	31.930	0.099	
DSB + effect of ∆_Rmax-R0_	26	27.745	0.009	
DSB + effect of CPB duration	27	22.443	< 0.001	
STEP 2				
DSB + effect of CPB duration + … effect of ∆_Rmax-R0_	26	17.574	< 0.001	− 4.869*

*P < 0.05, ∆(− 2LL) > 3.84. *CPB* cardiopulmonary bypass, *DSB* difficult separation from bypass, *− 2LL* objective function (− 2Log(Likelihood)), *LRT* Likelihood Ratio Test, *R*_*0*_ baseline mAP/mPAP ratio, *R*_*max*_ peak mAP/mPAP ratio, ∆(− 2LL), decrease in objective function, *∆*_*Rmax-R0*_ magnitude of peak response.

## Discussion

This is the largest report on detailed PK/PD of inhaled milrinone in cardiac surgery attempting to characterize inhaled milrinone concentration-effect relationship. When the mAP/mPAP ratio (R) was used as PD marker, magnitude of peak response before CPB (∆_Rmax-R0_) and CPB duration were both associated with DSB, suggesting that the former may represent a potential prognostic tool for DSB. The ∆_Rmax-R0_ represents the intensity of the pulmonary antihypertensive effect. The absence of a response might indicate a much more severe and irreversible pulmonary hypertension that may have prognostic value.

Given milrinone small molecular size (MW: 211.2), lipid solubility (log P: 1.17), as well as the large and well-perfused surface area provided by the lungs^30^, absorption process through the pulmonary route was expected to be extremely rapid (almost instantaneous)^31^. Indeed, many small molecules have pulmonary bioavailability approaching 100%^32–35^, which can be attributed to rapid pulmonary absorption and lower drug-metabolizing activity compared to the oral route^36–38^. After inhaled prochlorperazine, for example, superimposed plasma concentration–time profiles were observed after inhalation of a thermally generated aerosol or intravenous administration in both anesthetized dogs^39^ and humans^40^. Accordingly, a two-compartment model with a zero-order input was deemed adequate. In agreement with reports on mesh nebulizers^41^, milrinone treatment time varied greatly amongst our patients (10–30 min).

Both non-compartmental analysis and compartmental analyses yielded similar PK parameters and agreed with those reported after IV administration in congestive heart failure patients^42,43^ and patients undergoing cardiac surgery^44–46^, suggesting a rapid and complete pulmonary absorption of the estimated inhaled dose of milrinone in our patients. This observation was also reported by others when comparing PK with those obtained in congestive heart failure patients^44^ or when milrinone was administered before vs after CPB in cardiac patients^47^.

Time-specific single point measures of the intensity of effect represented by R_0_, R_max_, R_post-CPB_, as well as ∆_Rmax-R0_ have already been used as hemodynamic endpoints in cardiac surgery for patients with PH^11^. In our patients, the mean increase in R_post-CPB_ was not significant at the end of CPB when compared to R_0_ (*P* = 0.358). At this time-point, it is difficult to clearly distinguish the effect attributable to milrinone residual pharmacological effect from that induced by hemodynamic changes associated with CPB weaning. It is worth pointing out that both R_max_ and R_post-CPB_ were opened-chest measures while R_0_ was determined at closed-chest. For instance, mean value for R_post-CPB_ was 2.70 when measured after chest closure compared to 2.27 before chest closure. Thus, estimation of ∆_Rmax-R0_ mean value remains conservative and may have been higher if R_max_ could have been taken under closed-chest conditions. A higher degree of significance for the net maximal effect would be expected under closed-chest conditions. It was felt that, rather than looking at separate measurements over time, a more accurate estimate of the overall effect would be obtained by integrating effect over time^48^ and that, especially in presence of PD fluctuations^49^. Therefore, AUEC was used to evaluate the net PD response. A linear relationship between milrinone systemic exposure (AUC) and the corresponding pharmacologic effect exposure (AUEC) during inhalation would represent the first step towards establishment of a potential proof of concept. Such a relationship has recently been explored in a subset of patients from a randomized controlled trial with very limited PK and PD data^29^ and appeared worth pursuing with a more extensive approach.

Keeping in mind that the overarching goal is to obtain a readily accessible PD marker that would adequately reflect milrinone overall effect during the inhalation period, the significant correlation observed between AUEC and ∆_Rmax-R0_ (single point) suggests that the overall net effect is in agreement with the magnitude of peak effect (end of inhalation). Accordingly, non-responders showed both low AUEC and low ∆_Rmax-R0_ values as also observed in a clinical trial^29^. Other studies on inhaled milrinone administered prior to CPB in cardiac surgery have also observed 18–26% of non-responders amongst their population of pulmonary hypertensive patients^11,26,28^. Indeed, chronic hypoxia and vascular remodeling is assumed to result in secondary and in some cases fixed pre-capillary PH, which is an independent predictor of mortality^50^.

Finally, as the occurrence of DSB represents the major clinical endpoint for procedures requiring CPB, several potential predictors were explored using a logistic univariate regression model. AUEC was not retained mostly because these values are not readily computed before CPB. Single point PD markers readily available prior to CPB weaning (R_0_, R_max_ and ∆_Rmax-R0_) were considered because they are more pragmatic. Only the magnitude of peak response (∆_Rmax-R0_) and CPB duration remained in the final model. Our results are consistent with prior studies suggesting that inhaled milrinone administered prior to CPB would have a protective effect in pulmonary hypertensive patients^11,26^ by minimizing CPB-related inflammation^27^, preventing pulmonary endothelial dysfunction^25^ and facilitating separation from CPB^28^. The absence of a response might indicate a much more severe and irreversible pulmonary hypertension that may have prognostic value as suggested by a recent study^51^. As for CPB duration, it was already known to be a strong risk factor of DSB^28^. In addition, many other factors are likely to have a role in the etiology of DSB^1,52^.

The major limitation of this study was the impossibility of modeling each patient's whole set of concentration-effect data because PD data were often contaminated by surgical interventions. Moreover, inclusion criteria allowed a wide range of PH (sPAP 36–90 mmHg) and study population was not quite homogeneous (EuroSCORE 1.2-46.4). Milrinone dose may also have been suboptimal (taking into account the inhaled dose measured) and may require adjustments in further dose-ranging studies. In absence of rich data PK/PD analysis, our sample size may not have been sufficient. Despite this, the magnitude of peak pharmacological response (∆_Rmax-R0_) and CPB duration were both found to be associated with DSB.

In addition, it is known that the amount of air embolism following cardiac surgery can result in right ventricular failure which can only be quantified using transcranial Doppler^52,53^ which was not available at the time of the study. The amount of air is unpredictable and could explain why pre-CPB inhaled agents might not always prevent difficult separation from CPB. However, three studies using combined inhaled epoprostenol and inhaled milrinone (iE&iM), we observed that easier separation from CPB was also associated with a significant response to iE&iM treatment observed before CPB^51,54^ and reduced inotropic support after CPB^55^. In one of the study^51^, a higher proportion of non-responders had difficult separation from CPB and required intravenous inotropic drug support compared to responders. Use of intravenous inotropes after CPB was lower in responders to treatment (8.1% vs 27.6%; *P* = 0.0052). An increase of 20% in the mean arterial pressure to mPAP ratio was used to indicate a positive response to iE&iM. Another limitation of our study is the absence of a control group. A control group with intravenous milrinone would have been useful to demonstrate the hypotensive sparing effect of inhaled milrinone as supported by 4 small, randomized trials comparing inhaled versus intravenous administration^24,56–58^. The inhaled route results in a more slow release of milrinone into the systemic circulation and leads to reduced peak dose as we observed compared to intravenous administration^47^. This peak dose of milrinone is likely responsible for hypotension. Although a control group is rarely included in PK/PD studies, in this population other factors may influence R between the measurements of R_0_, R_max_ and R_CPB_. We cannot definitively establish a causal relationship between inhaled milrinone and changes in R. R_max-R0_ may reflect a more complex responsiveness of the pulmonary circulation to inhaled milrinone. Other factors could also influence our results such as limited duration of action or insufficient number of patients.

In summary, this is the first study reporting rich PK and PD data obtained after inhalation of milrinone in cardiac surgical patients. After mesh nebulization, milrinone absorption was extremely rapid and systemic levels remained within the therapeutic range. Both peak concentrations and maximum effects were observed at the end of inhalation. Comparison of respective milrinone AUC and AUEC before CPB provided preliminary evidence of a proof of concept for the use of the mAP/mPAP ratio before CPB as a promising PD marker. The magnitude of peak pulmonary circulatory response (∆_Rmax-R0_) may be a predictor of DSB. Further randomized controlled studies are required to confirm these findings (NCT05450328).

## Materials and methods

### Patients

After approval by the institutional research ethics committee (ICM 06-888; August 5, 2008) in accordance with the *Enoncé de politique des trois conseils (EPTC2)* and the Declaration of Helsinki, and with permission from Health Canada (non-objection letter, ref. 108851; November 2, 2006), the study was registered in ClinicalTrials.gov (ref: NCT01725776). Written informed consent was obtained from 28 patients having preoperative PH and scheduled for elective cardiac surgery under CPB. Patients were considered having PH if either one of the following conditions was met before surgery: systolic pulmonary artery pressure (sPAP) > 35 mmHg or mPAP > 25 mmHg^59^. Patients with hemodynamic instability prior to surgery were excluded. Procedures were classified as coronary revascularization, valvular surgery or complex, defined as a combination of two or more different procedures. The EuroSCORE II was calculated for each patient^60^.

#### Surgical procedure

Patients were premedicated with 1–2 mg lorazepam orally 1 h before surgery and received 0.1 mg kg^−1^ morphine intramuscularly before entering the operating room where midazolam was given (0.01–0.05 mg kg^−1^ intravenously) as needed for patient comfort. Usual monitoring was installed, including a 5-lead electrocardiogram, pulse oximeter, peripheral venous line, radial arterial line, 3-lm catheter, and fast-response thermodilution pulmonary artery catheter. Anesthesia was induced with 1 μg kg^−1^ sufentanil and 0.04 mg kg^−1^ midazolam, and muscle relaxation achieved with 0.1 mg kg^−1^ pancuronium. After tracheal intubation, anesthesia was maintained with 1 μg kg^−1^ h^−1^ sufentanil and 0.04 mg kg^−1^ h^−1^ midazolam. Intravenous fluids (0.9% normal saline) were administered (7 cc kg^−1^ h^−1^) during surgery and titrated according to blood pressure and central venous pressure. A transesophageal echocardiography (TEE) omniplane probe was inserted. Institution of CPB was performed using ascending aortic cannulation and bi-caval or double stage cannulation of the right atrium. Intermittent (4:1) blood cardioplegia was administered during CPB; induction and temperatures ranged from 15 to 29 °C. For coronary revascularizations, systemic temperature was allowed to drift to 34 °C, valvular surgeries and complex procedures to 32–34 °C. Weaning from CPB was undertaken after rewarming to a systemic temperature > 36 °C.

#### Drug administration

After induction of anesthesia, a TEE exam was conducted. Then, a 5 mg dose (50–80 µg kg^−1^) of milrinone (Milrinone Lactate 1 mg ml^−1^ (base); Pharmaceutical Partners of Canada Inc., Richmond Hill, ON, CAN) was administered by inhalation before initiation of CPB, using a mesh nebulizer (Aeroneb Professional Nebulizer System; Aerogen Ltd., Galway, Ireland). The dosage was based on previous clinical trials^11,29^. The nebulizer was attached to the inspiratory limb of the ventilator Y-connector near the endotracheal tube. Milrinone solution was placed into the nebulizer cup and inhalation was continued until aerosol production was deemed complete after gentle tapping of the device.

### Pharmacokinetic study

#### PK sampling

Serial arterial blood draws (5 ml) were obtained before inhalation (blank; 0 min), during inhalation (2, 5, 10, 15 min) and after the end of inhalation (0, 3, 6, 9, 15, 30, 60, 90, 120, 180, 240, 360, 480, 600 min). Two samples were also obtained after initiation of CPB (2 min) and after weaning from CPB (2 min). Blood samples were kept on ice for a short period of time and centrifuged. Plasma was immediately flash-frozen on dry ice and stored at − 80 °C. Milrinone plasma concentrations were determined by high performance liquid chromatography (HPLC) using tandem mass spectrometry detection^61^. The lower limit of quantification (LLOQ) was 0.3125 ng ml^−1^ with mean intra-assay (n = 6) and inter-assay (n = 10) precisions < 12%, expressed as coefficients of variation (CV%).

#### Inhaled dose

In the case of milrinone, the molecule being almost exclusively (> 95%) excreted unchanged or conjugated in urine, measurement of total urinary excretion allows for a realistic approximation of the inhaled dose^42,62^. For fifteen patients, complete 24 h-urine collections were therefore used for external validation. Total (conjugated and unconjugated) urinary concentrations of milrinone were measured by HPLC using ultraviolet detection^63^. In vivo experiments were also carried out by measuring the exhaled dose and the residual dose in the nebulizer cup. The total dose recovered was estimated by summing individual recoveries determined in vivo (including urinary excretion) and mean recovery previously obtained in vitro for components that could not be disconnected during cardiac surgery (*i.e*., nebulizer T-piece, Y-connector and endotracheal tube)^64^. Since complete 24-h urine collection is often difficult to ascertain in a clinical setting, a back-calculated approach for the estimation of the inhaled dose was used by subtracting the total dose recovered (individual in vivo and in vitro mean values) from the nominal dose administered (5 mg). This back-calculated value was then compared with the cumulative amount of milrinone recovered in urine for the same patient and considered for PK analysis.

#### PK analysis

Milrinone absorption process through pulmonary route is extremely rapid after inhalation^35^. A two-compartment model with zero-order input rate during nebulization and elimination from the central compartment was fitted to individual milrinone plasma concentration–time profiles, after standard verification of its adequacy using the Akaike information criterion. Point estimates and PK parameters were optimized for individual data using a standard minimization method (Gauss–Newton, Levenberg and Hartley) and a weighting function of 1/ŷ (where ŷ is the predicted concentration) was applied. Parameters including peak concentration (C_max_), peak time (T_max_), coefficients of bi-exponential equation describing disposition curve (A, B), fast distribution and elimination rate constants (α, β), total body clearance and apparent volume of distribution expressed as a function of bioavailability (Cl/F, V/F) were determined using WinNonlin® Version 5.3 software (PK Model 10, Pharsight Corp., Mountain View, CA, USA). Relationship between milrinone systemic exposure and nebulization rate was also explored.

For most routes of administration, the dose given to a patient is assumed to be completely delivered. This is often not the case for the pulmonary route and even less for the inhaled dose which represents the fraction of the nominal dose that ultimately reaches the distal end of the endotracheal tube. In the context of cardiac surgery (in vivo setting), milrinone inhaled dose could not be directly measured and was estimated using a back-calculated approach based on combined in vivo and in vitro data accounting for quantifiable and non-quantifiable losses within the respiratory apparatus, respectively. Since milrinone is almost completely excreted unchanged, urinary data (complete 24-h urine collection in a subset of 15 patients included herein) served as an external validation (Supplementary Table S1). According to this approach, mean total dose recovery was estimated as 95.3% of the 5 mg nominal dose, which included the inhaled dose, exhaled dose, residual and wasted doses within the nebulizer and delivery system. For these reasons, individual back-calculated inhaled doses were estimated and used for PK analyses.

### Pharmacodynamic study

#### PD markers

Hemodynamic parameters including mAP and mPAP were continuously monitored and data recorded at 1- and 15-min intervals during the pre- and post-CPB period, respectively. The mAP/mPAP ratio (R) was later calculated and used as our PD marker mostly on the basis of sounded evidence for its prognostic value as the best predictor of perioperative complications in cardiac surgery^5–10,14^. Previous studies^11,12^ and case report^13^ described how increases in the ratio following administration of inhaled agents in patients are associated with improvement of the right ventricular function. The mAP/mPAP ratio was also correlated with the eccentricity index (which reflects the intraventricular deformation resulting from PH^14^) and identified as a potential PD marker^29^. A normal value for mAP/mPAP ratio is generally expected to be greater than 4; thus, lower values are good indicators of the severity of PH. Thus, in patients under general anesthesia and in absence of surgical stress, the mAP/mPAP ratio should change proportionally to any alteration of PH. Surgical interventions, whenever possible, were avoided during the inhalation period. For each patient, closed-chest baseline mAP/mPAP ratio (R_0_) was determined from measures collected within 10 min immediately before inhalation (both mAP and mPAP had to be stable by visual inspection for at least 3 min). As baseline values are of paramount importance for PD noncompartmental analysis, R_0_ values were rigorously determined by using the average value obtained from two independent experimenters. This approach for baseline characterization was carried out during the pre-inhalation period and before any intervention (TEE, legs raising, skin incision, or other surgical procedures).

Both open-chest peak mAP/mPAP ratio (R_max_) and post-CPB mAP/mPAP ratio (R_post-CPB_) were also considered as single point PD markers. Another PD marker frequently used in our clinical setting, that is the magnitude of peak response (∆_Rmax-R0_), was also calculated. A one-way repeated measures analysis of variance (ANOVA) (SigmaPlot™ Version 11.2, Systat Software Inc., San Jose, CA, USA) was used to compare R_0_, R_max_ and R_post-CPB_. Lastly, the relationship between these PD markers and DSB (clinical endpoint) was also explored.

#### PK/PD analysis

Milrinone concentration–response relationship was analyzed by correlating patients’ respective area under the plasma concentration–time curve (AUC) and area under the response-time curve (AUEC) calculated using the linear trapezoidal rule. For the calculation of AUEC, both positive and negative fluctuations from the predetermined baseline response (R_0_; reference value) were taken into account during integration. Summation of all positive and negative partial AUEC yielded a net AUEC (NCA Model 220, Pharsight Corp., Mountain View, CA, USA). The AUC-AUEC relationship was investigated during the inhalation period (from 0 min until the end of inhalation). First, correlation was evaluated using all patients. The AUEC-intercept given by linear regression was considered to be the minimum threshold for response and considered as cut-off for determining responders. Then, correlation was re-evaluated in responders only. Finally, correlation between AUEC and ∆_Rmax-R0_ was verified, and consistency of results confirmed.

#### Clinical exploratory endpoint

The occurrence of DSB is considered as an important clinical endpoint in cardiac surgery. Two definitions were used to stratify the severity in weaning from CPB and were exclusively based on the type of support used from the end of CPB until the end of the surgery^1^. Easy separation from bypass was defined as either no support needed or only one vasoactive (norepinephrine, phenylephrine, vasopressin) or inotropic (dobutamine, milrinone, epinephrine) agent being used. Difficult separation from bypass (DSB) was defined as the requirement for at least both vasoactive and inotropic agents or also defined as ≥ 1 failure of the first weaning attempt or the requirement for an intra-aortic balloon pump or a ventricular assist device to leave the operating room. As a secondary exploratory endpoint, we explored a plausible relationship between response to inhaled milrinone (selected single point PD drivers) and DSB. Because PH was identified as one of the most important hemodynamic predictor and risk factor for DSB^3,4^, a positive response to inhaled milrinone in attempt to control PH was considered a potential predictor of DSB. Since the exploratory objective was to identify potential prognostic variables for DSB, variable selection was also based on clinical relevance that is prior knowledge of the pathophysiology related to CPB and factors susceptible to impact on its outcome. Logistic regression was carried out to identify factors independently associated with DSB. Several potential predictors were explored (EuroSCORE II, R0, Rmax, ∆Rmax-R0 and CPB duration). Simple and multiple logistic regressions were performed with stepwise selection (SigmaPlot™ Version 11.2, Systat Software Inc., San Jose, CA, USA) were used to develop a multivariate predictor of DSB.

## Supplementary Information

Supplementary Tables.

## Data Availability

All data will be available on reasonable request to the corresponding author.

## Abbreviations

α: Distribution rate constant
β: Elimination rate constant
ANOVA: Analysis of variance
AUC: Plasma concentration–time curve
AUEC: Area under the response–time curve
Cl: Total body clearance
Cmax: Peak plasma concentration
CPB: Cardiopulmonary bypass
DSB: Difficult separation from bypass
EuroSCORE II: European system for cardiac operative risk evaluation
F: Bioavailability
mPAP: Mean pulmonary artery pressure
PD: Pharmacodynamic
PH: Pulmonary hypertension
PK: Pharmacokinetic
R: Ratio
R_0_: Closed-chest baseline mAP/mPAP ratio
R_max_: Open-chest peak mAP/mPAP ratio
R_post-CPB_: Post-CPB mAP/mPAP ratio
∆_Rmax-R0_: Magnitude of peak response of the mAP/mPAP ratio
Tmax: Peak time
V_c_: Apparent volume of distribution of central compartment
V_ss_: Apparent volume of distribution at steady-state
