# Supplementary material for: Inhaled milrinone in cardiac surgical patients: pharmacokinetic and pharmacodynamic exploration
Source: Sci Rep. 2023 Mar 2;13:3557. doi: 10.1038/s41598-023-29945-7 (PMC9981759; doi:10.1038/s41598-023-29945-7)
Supplement: Supplementary file 1 — Supplementary Tables. [file 41598_2023_29945_MOESM1_ESM.pdf]

Inhaled milrinone in cardiac surgical patients: pharmacokinetic and pharmacodynamic exploration

Anne Quynh-Nhu Nguyen, Ph.D.[1], André Y. Denault, M.D., Ph.D.[2]*,

Yves Théoret, B.Pharm., Ph.D.[3], France Varin, B.Pharm., Ph.D.[1]*

SUPPLEMENTARY TABLES X2

Supplementary Table S1. *In Vitro* Experiments for Milrinone Dose Recovery.

	Mesh Nebulizer		
	n	%	
Inhaled dose (24-h urine collection)	15	28.5	(8.2)
Exhaled dose	14	26.4	(6.5)
Residual dose (nebulizer cup)	14	3.5	(1.3)
Wasted dose (nebulizer T-piece)†	3	18.2	(4.4)
Wasted dose (Y-connector + endotracheal tube)†	3	21.4	(3.5)
**Total dose recovered**		**95.3**	**(10.7)**

All values are mean (standard deviation) and expressed as percentage (%) of nominal dose (5 mg).

$P<0.05$. †Data obtained from *in vitro* experiments (Nguyen et al. *Sci. Rep*. 10, 2069 (2020)).

Supplementary Table S2. Milrinone PK Parameters after Non-Compartmental Analysis.

$D_{inhaled}$† mg		T_{max} min		C_{max} ng·ml^{-1}		k_{el} min^{-1}		Cl/F L·h^{-1}·kg^{-1}		V_d/F L·kg^{-1}	
1.52	(0.32)	20	(4)	125	(38)	0.0045	(0.0017)	0.11	(0.06)	0.46	(0.29)

All values are mean (standard deviation). †Backcalculated inhaled doses estimated according to *in vivo* and *in vitro* results (see Materials and Methods for details). C_{max}, peak concentration; Cl, clearance; $D_{inhaled}$, inhaled dose; F, bioavailability; k_{el}, elimination rate constant; PK, pharmacokinetic; T_{max}, peak time; V_d, volume of distribution.
